# On the (Un)greenness of Biocatalysis: Some Challenging Figures and Some Promising Options

**DOI:** 10.3389/fmicb.2015.01257

**Published:** 2015-11-12

**Authors:** Pablo Domínguez de María, Frank Hollmann

**Affiliations:** ^1^Las Palmas de Gran CanariaLas Palmas, Spain; ^2^Biocatalysis and Organic Chemistry Group, Department of Biotechnology, Delft University of TechnologyDelft, Netherlands

**Keywords:** biocatalysis, organic chemistry, green chemistry, solvents, organic synthesis

## Abstract

Biocatalysis is generally regarded as a “green” technology. This statement is justified by the mild reaction conditions, the use of aqueous reaction media—with water as the paradigm of green solvents—, and the renewable nature of the biocatalysts. However, researchers making these statements frequently do not take into account the entire picture of their processes. Aspects like water consumption, wastewater production, titers, and metrics of the (diluted?) biocatalytic processes are important as well. With those figures at hand, many biocatalytic reactions do not appear so green anymore. This article critically discusses some common wrong assumptions given for biocatalytic approaches, with regard to their environmental impact, and actual greenness. Some promising biocatalytic approaches, such as the use of biphasic systems involving biogenic solvents, deep-eutectic-solvents (and biogenic ionic liquids), water-free media, solvent-free processes, are briefly introduced, showing that enzyme catalysis can actually be a robust sustainable alternative for chemical processes.

## Introduction

Through the development of Molecular Biology, biocatalysis has emerged as a technology for organic synthesis, complementing other areas of catalysis. Key-assets of biocatalysis are the often observed high selectivity of enzymes—either isolated enzymes or whole cells—, displaying regio-, enantio-, or chemo-selectivities, together with their production *via* fermentation in large quantities.

In addition, environmentally-friendly conditions are applied in enzyme-catalysis, performed at ambient pressure and temperature (albeit biocatalysts from extremophiles can also be successfully used with *harsher* reaction parameters). On this basis, the “environmental benignity” is often used as a strong “selling argument” for biocatalysis, especially when compared with other branches of catalysis. Unfortunately, there is commonly a huge gap between the mere statement of such green claims, and critical and honest quantitative studies that may support this with scientific evidences. Actually, most of the published articles dealing with biocatalysis take the greenness for granted, without providing consistent data. This vague tendency actually represents more a problem than a solution for biocatalysis, as many chemists become reluctant in using enzymes, especially when a detailed investigation of the reaction parameters of many biocatalyzed reactions leads to rather questionable ecological measurements.

Aiming at contributing to this debate, the goal of this article is to discuss some of the green claims frequently associated with biocatalysis, and to illustrate why the desired ecological footprints are actually not reached. Being constructive, some possible solutions and options for sustainable biocatalysis are highlighted.

## Water as solvent: scope and limitations

Many publications state that water is an environmentally-benign solvent. Indeed, water exhibits beneficial properties, it is available in sufficient quality and quantity for chemical transformations performed at small-to-moderate scale—especially when non-potable or residual water is (re)used—, is non-hazardous and does not present problems of flammability or explosion risks associated to other organic solvents.

However, one major disadvantage of water as solvent lies within its high polarity. Hydrophobic reagents—synthetically, often the most interesting and desirable ones—are poorly soluble in aqueous media. Unfortunately, this challenge is often met by adjusting the reagent concentration to the lower millimolar range. In other words, a typical biocatalytic reaction mixture (claimed to be Green Chemistry) contains actually more auxiliary reagent than product (Table 1).

**Table 1:** **Typical reagent conditions for biocatalysis using poorly water soluble reagents**.

**Component**	**Typical concentration [mol L^−1^]**	**Mass ratio (auxiliary to product) [kg kg^−1^]**
Water	55	500
Buffer^a^	0.05	2
Enzyme^b^	1 × 10 ^−6^	0.04
Product^c^	0.010	1

a *50 mM potassium phosphate*.

b *a M_w_ of 40 kDa*.

c *an average M_w_ of 200 g mol^−1^*.

From Table 1 it is straightforward to calculate the volume of solvent (water) needed to deliver 1 Kg of a product. Furthermore, another fact is that the reaction medium—even after extensive downstream processing—will still contain trace amounts of reagents and catalysts, and therefore it will have to be considered as waste product, which has to be treated prior to disposal. Any treatment will require additional reagents and energy (and expenses), thereby contribute negatively to the environmental impact of the reaction. Remarkably, very few publications actually consider recycling of the aqueous reaction mixture. As an example of this, Greiner et al. demonstrated that recycling the aqueous reaction mixture can reduce the E-factor (Kg of waste produced per Kg of product) by more than 10-fold (Leuchs et al., 2013). This impressive reduction deserves further investigation to extend it to other biocatalytic applications in aqueous media. The set-up of membrane reactors, immobilized catalysts, as well as overcome issues derived from cofactor regeneration, may also add value to these premises.

Another facet of diluted biocatalysis at low substrate loadings is the poor economic performance. Stirring a mixture containing only a few grams per liter can only be profitable if the product is of very high value, and provided that no other synthetic alternatives exist. To some extent this explains why biocatalysis is still frequently considered as “too inefficient” and “too expensive”: Being honest, the term “diluted aqueous solutions” does not actually match very well with “robust industrial processes,” but rather with “academic curiosity.” As a rule of thumb for fine chemicals, processes should run at preferably > 50 g substrate L^−1^, and more preferably at > 100 gL^−1^.

## Overcoming the solubility issue. tips and tricks reported in the literature

To overcome the above-discussed challenges several strategies have been put forth. One of them is using water-miscible co-solvents to increase the overall reagent concentration of biocatalytic reactions. Popular water-miscible solvents comprise (amongst others) alcohols such as methanol, ethanol, *tert*-butanol, or *iso*-propanol. These co-solvents are particularly useful for enzymes like hydrolases, oxidoreductases, or lyases. Advantageously in the case of alcohol dehydrogenase (ADH)-catalyzed reduction reactions, they may serve two functions: (1) as co-solvent and (2) as co-substrate (sacrificial electron donor). Moreover, by using alcohol co-solvents in excess it is possible to shift unfavorable equilibria to the product formation. An important point herein is to consider the biocompatibility of the enzyme with the intended co-solvents.

However, the major drawback when using water-miscible co-solvents occurs with enzymatic reactions that may be prone to product inhibition (as frequently observed with ADHs). The rate of the reaction will decrease with conversion, leading to critically slow reactions or even complete cease at non-satisfactory product concentrations. Another issue is the sometimes unpredictable effect on the biocatalyst activity and stability, as the logP value does not always serve as prediction property for biocatalysis (Villela Filho et al., 2003). Hence optimal reaction conditions will have to be defined for every new biocatalyst and reaction. Nevertheless, despite these drawbacks, in many biotransformations the use of these cost-effective and biodegradable water-miscible co-solvents represents an excellent alternative to enhance substrate loadings while leading to outstanding enzymatic enantioselectivities and productivities. Thus, it must be always considered for optimization of biocatalytic processes with industrial vistas.

In addition to these water-miscible co-solvents, the use of hydrophobic solvents is well-known, enabling the so-called two liquid phase systems (2LPS). A water-immiscible solvent serves as substrate reservoir and product sink, and a simplified work-up is reached at the same time, in which the product is directly obtained from the organic phase upon distillation. Using this set-up, the overall substrate loadings of various reactions could be raised significantly. For example the whole-cell production of (*S*)-4-chloro-3-hydroxybutanoate ethyl ester from the corresponding ketone could be improved significantly by using butyl acetate as second organic phase. The overall substrate loading was significantly increased whereas the E-factor decreased from 520 (in the dilute aqueous system) to 8 (in the 2LPS) (Wu et al., 2009; Ye et al., 2010). Analogously, Evonik has developed an oxidoreductase platform—designing recombinant whole-cells overexpressing two enzymes simultaneously—, using MTBE (*tert*-butyl methyl ether) as second phase, with high productivities of > 150 g L^−1^ and excellent enantioselectivities (Gröger et al., 2006a). To complement competitive biocatalysis with improved environmental footprints, recently the use of biomass-derived 2-methyl tetrahydrofuran (2-MeTHF) as second phase (also as co-solvent at low proportions, < 5% v/v) has been proposed (Pace et al., 2012), combining high biocatalytic productivities, substrate loadings, and acceptable waste production.

2LPS also offer other advantages to overcome difficulties associated with unfavorable equilibria of enzymatic reactions, which can be shifted to the adequate side by smart use of the organic phase. Likewise, in the case of reactions and catalysts prone to product inhibition, the extraction of the product from the reactive phase can lead to significant accelerations of the reaction. Kragl et al. demonstrated that the ionic liquid [BMIM][(CF_3_SO_2_)_2_N] efficiently removes acetone from the aqueous phase and thereby diminished its inhibitory effect on ADH-catalyzed reductions (Eckstein et al., 2004). Similarly, another interesting application of 2LPS uses hydrophobic organic phases to selectively extract aldehydes. Thus, “over-oxidation” of alcohols to carboxylic acids can be prevented by simply separating the reactive intermediate from the catalysts (Bühler et al., 2000, 2002, 2003; Villa et al., 2002; Aksu et al., 2009). Likewise, prevention of “over-reduction” of carboxylic acids to the corresponding alcohols can be achieved (Gandolfi et al., 2001). Finally, a “classic application” of 2LPS lies in the protection of hydrolysis-prone products such as epoxides (Panke et al., 2002; Hofstetter et al., 2004; Park et al., 2006; Toda et al., 2012; Ni et al., 2014) or lactones (Schmid et al., 2001) by separating them from the aqueous layer through *in situ* extraction.

However, 2LPSs are limited due to sluggish phase transfer rates slowing down the overall reaction rate. Since the phase transfer rate (next to other factors) directly correlates with the surface area, emulsions are an attractive means to circumvent this limitation. But the demanding mechanical conditions can lead to shear stress-induced inactivation of the biocatalysts (Karande et al., 2011; Churakova et al., 2013; Kara et al., 2013). Herein, the set-up of homogeneous mini-emulsions has proved to be an alternative for industrial processes, as Evonik has shown in the synthesis of β-amino acids (e.g., β-phenyl alanine) using lipases (Gröger et al., 2006b).

## Non-conventional media: from neoteric solvents (ILs and DES) to water-free and solvent-free “neat” systems

Another approach for industrially-sound biocatalysis is the so-called non-conventional media, namely performing enzymatic reactions in non-aqueous solutions, in which the solubility of the substrates does not represent a hurdle. Herein, the challenge is to identify reaction media that may be enzyme-compatible and sustainable.

A first attempt to combine enzyme-compatible media with sustainability was using ionic liquids (ILs) as solvents. A number of academic applications of enzymes and whole-cells—including hydrolases, oxidoreductases, lyases, etc.—, performing outstandingly in such ILs were reported (Domínguez de María, 2012). However, despite ILs were initially regarded as green solvents (based on their low vapor pressure), it was realized that many ILs lead actually to environmental problems, toxicity, accumulation in the milieu, etc. Furthermore, their high boiling point obliged in many cases to perform product extractions using organic solvents—the ones that were actually intended to be removed.

**Figure 1:**
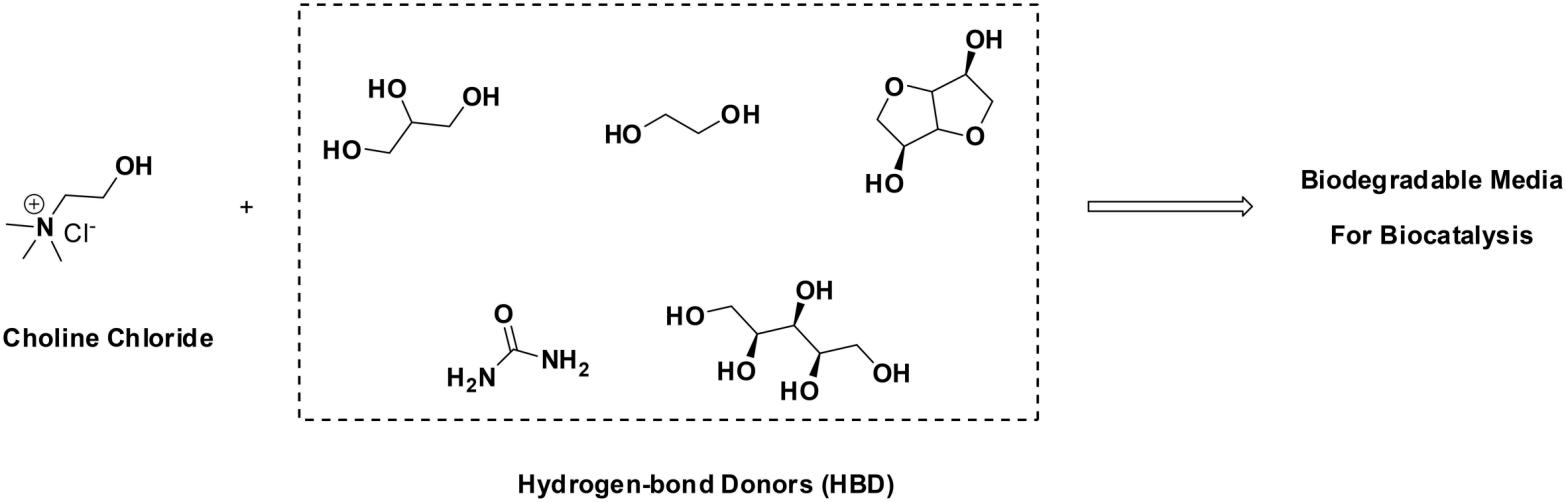
**Formation of biodegradable DES as promising environmentally-friendly solvents for biocatalysis (Domínguez de María, 2012; Maugeri et al., 2013; Maugeri and Domínguez de María, 2014a,b; Müller et al., 2015)**.

Attempting to combine the excellent features that ILs may have—negligible vapor pressure, enzyme-friendly, solubilizers, etc.—, with improved ecological prognoses, the use of Deep-Eutectic-Solvents (DES) has been put forth. DES are formed when quaternary ammonium salts (e.g., choline chloride) are combined with hydrogen-bond-donor molecules (HBDs, such as alcohols, amines, carboxylic acids, etc). HBDs generate a distortion in the crystalline structure of the quaternary ammonium salt, decreasing the melting point of the mixture, rendering solvents at room temperature. Outstanding examples are combinations of choline chloride with urea, glycerol, xylitol, isosorbide, etc. Having cost-effective and biodegradable components, DES pose promising features for sustainable chemistry (Figure 1). A number of enzymes and whole-cells have already been shown to be active in DES, covering hydrolases, oxidoreductases, lyases, etc (Domínguez de María, 2012; Maugeri et al., 2013; Maugeri and Domínguez de María, 2014a,b; Müller et al., 2015).

Another interesting approach for sustainable biocatalysis–reduced waste, high productivities, competitiveness—is represented by the set-up of biocatalytic reactions in neat substrates, in the absence of bulk water. Herein, the use of liquid substrates are advantageous due to the extremely high substrate loadings (as substrates act as “solvent” too), what leads to very high productivities. Several examples using whole-cells overexpressing oxidoreductases (de Gonzalo et al., 2007; Jakoblinnert et al., 2011) and lyases (Jakoblinnert and Rother, 2014) have been reported. Moreover, downstream processing is typically straightforward, as after filtration to recover the whole-cells, a simple distillation to remove reagents is enough to render the desired product. The neat-substrate approach represents a promising option to combine industrial level with highly promising environmental standards.

## Concluding remarks

Biocatalysis is one of the key-technologies en-route to making tomorrow's chemical industry cleaner and less energy demanding. However, biocatalysis is not green *per se*, and as any other technology it exhibits advantages and disadvantages. In recent years a common trend evolved to associate everything related to “bio” as green and environmentally friendly. In this contribution we show that the example of water as a green solvent for biocatalysis leads often to highly questionable statements. However, we also show that many creative solutions exist to overcome this issue: biogenic cosolvents, biphasic media, deep-eutectic-solvents, solvent-free or water-free media, or even multi-step catalytic processes. Many of those strategies lead to simpler and less pollutant downstream processing, another key-step for industrial biocatalysis. Based on a realistic and (semi-)quantitative analysis of the environmental impact, suitable solutions as outlined here for the solvent issue can be envisioned. Let's make biocatalysis become truly Green by replacing Green claims by quantitative data!

## Funding

FH gratefully acknowledges support by the European Union (ERC Consolidator Grant No. 648026).

### Conflict of interest statement

The authors declare that the research was conducted in the absence of any commercial or financial relationships that could be construed as a potential conflict of interest.
